# Mapping N- to C-terminal allosteric coupling through disruption of a putative CD74 activation site in D-dopachrome tautomerase

**DOI:** 10.1016/j.jbc.2023.104729

**Published:** 2023-04-18

**Authors:** Emily Chen, Vinnie Widjaja, Gregory Kyro, Brandon Allen, Pragnya Das, Varsha M. Prahaladan, Vineet Bhandari, Elias J. Lolis, Victor S. Batista, George P. Lisi

**Affiliations:** 1 Department of Molecular Biology, Cell Biology, & Biochemistry, Brown University, Providence, Rhode Island, USA; 2 Department of Chemistry, Yale University, New Haven, Connecticut, USA; 3 Section of Neonatology, Department of Pediatrics, Cooper University Hospital, Camden, New Jersey, USA; 4 Department of Pharmacology, Yale University School of Medicine, New Haven, Connecticut, USA

**Keywords:** NMR, allostery, cytokine, protein dynamics, receptor activation

## Abstract

The macrophage migration inhibitory factor (MIF) protein family consists of MIF and D-dopachrome tautomerase (also known as MIF-2). These homologs share 34% sequence identity while maintaining nearly indistinguishable tertiary and quaternary structure, which is likely a major contributor to their overlapping functions, including the binding and activation of the cluster of differentiation 74 (CD74) receptor to mediate inflammation. Previously, we investigated a novel allosteric site, Tyr99, that modulated N-terminal catalytic activity in MIF through a “pathway” of dynamically coupled residues. In a comparative study, we revealed an analogous allosteric pathway in MIF-2 despite its unique primary sequence. Disruptions of the MIF and MIF-2 N termini also diminished CD74 activation at the C terminus, though the receptor activation site is not fully defined in MIF-2. In this study, we use site-directed mutagenesis, NMR spectroscopy, molecular simulations, *in vitro* and *in vivo* biochemistry to explore the putative CD74 activation region of MIF-2 based on homology to MIF. We also confirm its reciprocal structural coupling to the MIF-2 allosteric site and N-terminal enzymatic site. Thus, we provide further insight into the CD74 activation site of MIF-2 and its allosteric coupling for immunoregulation.

The human cytokine-like enzymes macrophage migration inhibitory factor (MIF) and D-dopachrome tautomerase (also called MIF-2) are broadly expressed immunoregulators (1). Elevated levels of MIF family proteins are implicated in inflammatory diseases such as asthma, acute respiratory distress syndrome, and arthritis, while antibody neutralization of MIF proteins attenuates inflammatory symptoms in animal models (2). Current research surrounding the therapeutic targeting of the MIF superfamily is limited by the fact that wholesale inhibition is undesirable due to their protective roles in innate immunity and host microbial responses. New efforts to locate and characterize allosteric sites within this superfamily is a promising avenue to regulate their disease-state function. Such an undertaking relies on detailed structural and dynamic information and is bolstered by the fact that several intervention points for MIF and MIF-2 are shared, including the transcriptional activation of proinflammatory factors upon binding to cluster of differentiation 74 (CD74), the recruitment of signaling subunit CD44 to the MIF-CD74 complex, the activation of extracellular signal-regulated kinases, and the recruitment of leukocytes (3, 4, 5). These conserved functions are thought to be attributed to the shared tertiary and quaternary structure of MIF and MIF-2. An evolutionarily conserved proline at the N terminus acts as the catalytic base for an enol-keto tautomerase activity of unknown biological significance in both MIF and MIF-2. However, the literature overwhelmingly supports a role for the MIF enzymatic site in its “cytokine activity,” namely the activation of CD74. Indeed, mutation of Pro1 abolishes enzymatic activity and diminishes CD74 activation *in vivo* (6, 7).

The ability of MIF and MIF-2 to toggle their activities has been attributed to allostery; the regulation of chemical function from spatially distant sites within the proteins. These remote effects may occur through intramolecular clusters of residues (*i.e.*, allosteric pathways) or through changes in shape mediated by alterations to intermolecular forces and may contribute separately or synergistically to changes in function. MIF-2 maintains classical hallmarks of allostery, including a multidomain structure with several ligand docking sites and a conformational equilibrium that is stabilized in discrete states by its binding partners. We previously demonstrated that a novel allosteric residue, Tyr99, modulated N-terminal catalytic activity in MIF through a pathway of dynamic crosstalk comprising intramolecular and intermolecular correlations (8). Disruption of the MIF N terminus revealed that allosteric residues in this region also diminished CD74 activation, which occurs at the C terminus. Additionally, we showed that N-terminal mutations in the MIF-2 homolog caused widespread nuclear magnetic resonance (NMR) perturbations and line broadening at its C terminus with a concomitant decrease of CD74 activation *in vivo* (9), though the CD74 activation site of MIF-2 has not been extensively mapped. At present, it is clear that the C terminus of MIF-2 “senses” the structural and dynamic effects of mutations at the N terminus, but whether allosteric crosstalk is reciprocal based on perturbations at the C terminus or whether the C terminus is a functional hub in MIF-2, is unknown and explored here. We introduced a series of mutations at the MIF-2 C terminus, located at the hypothesized site of CD74 activation and allosteric regulation. We used NMR spectroscopy, molecular dynamics (MD) simulations, and *in vitro* and *in vivo* functional assays to investigate whether the C terminus of MIF-2 indeed contributes to the allosteric pathway partially mapped in this system and explored previously in MIF.

## Results

### Disruption of the MIF-2 C terminus attenuates enzymatic activity

**Figure 1:**
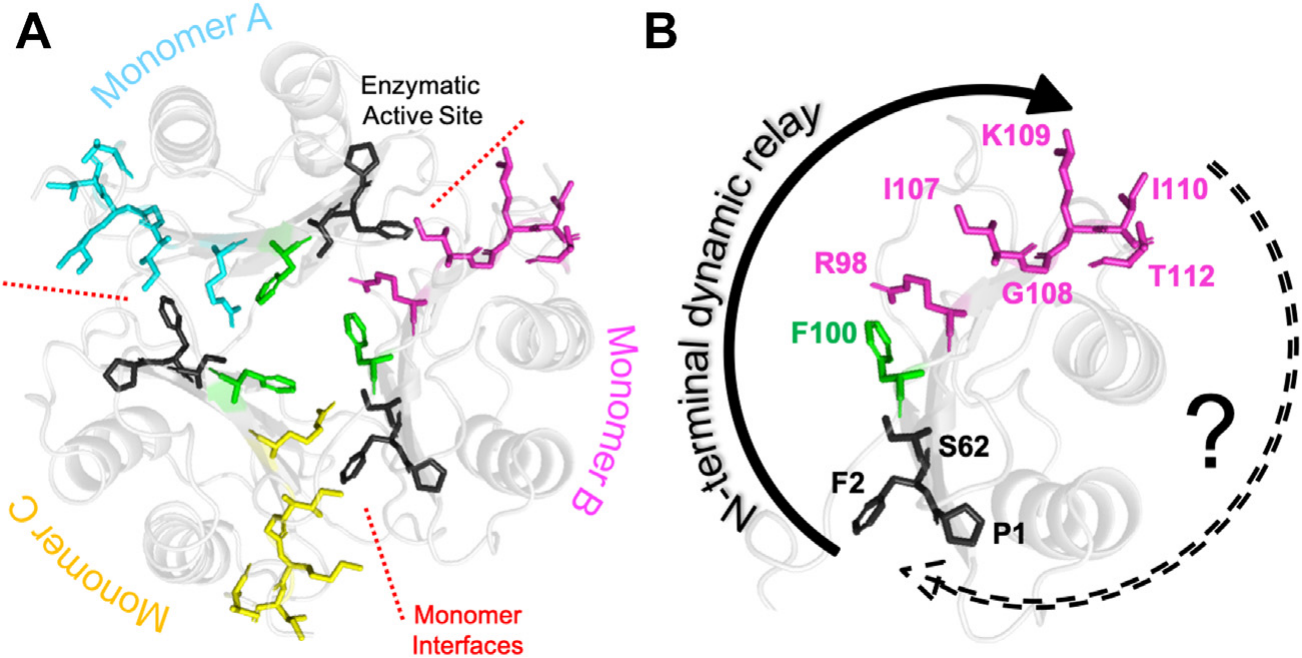
**Proposed MIF-2 allosteric pathway.** Residues involved in the dynamic relay from the catalytic site to the proposed CD74 activation site are shown as *sticks* on the MIF-2 structure (PDB ID: 7MSE). *A*, quaternary structure of MIF-2 showing the symmetric trimer, its enzymatic active site and monomer interfaces, and the proposed allosteric network within each monomer. *B*, our prior work confirmed an “outgoing” dynamic and functional communication between the N and C termini (*solid arrow*). A reciprocal effect of C-terminal mutations on the N terminus is the subject of this study (*dashed arrow*). *Black*: previously investigated N-terminal residues; *green*: allosteric node Phe100; *magenta*: C-terminal residues under investigation for their involvement in MIF-2 allostery. CD74, cluster of differentiation 74; MIF-2, D-dopachrome tautomerase.

In our prior study of N-terminal mutations in MIF-2, we noted strong structural and dynamic coupling between the N terminus, solvent cavity at the trimer symmetry axis, Phe100 allosteric site (which is analogous to the Tyr99 allosteric site in MIF), and C terminus *via* NMR chemical shifts and relaxation parameters. We used this as a basis for our current work to (1) confirm allosteric reciprocity at the MIF-2 C terminus and (2) map the CD74 activation site in MIF-2, which has not been defined. We systematically mutated MIF-2 residues Arg98, Ile107, Gly108, Lys109, Ile110, and Thr112 that appear in the same spatial positions as those determined to regulate CD74 activation in MIF (Asn97, Val106, Gly107, Trp108, Asn109, and Ser111, Fig. 1) (6, 10).

**Figure 2:**
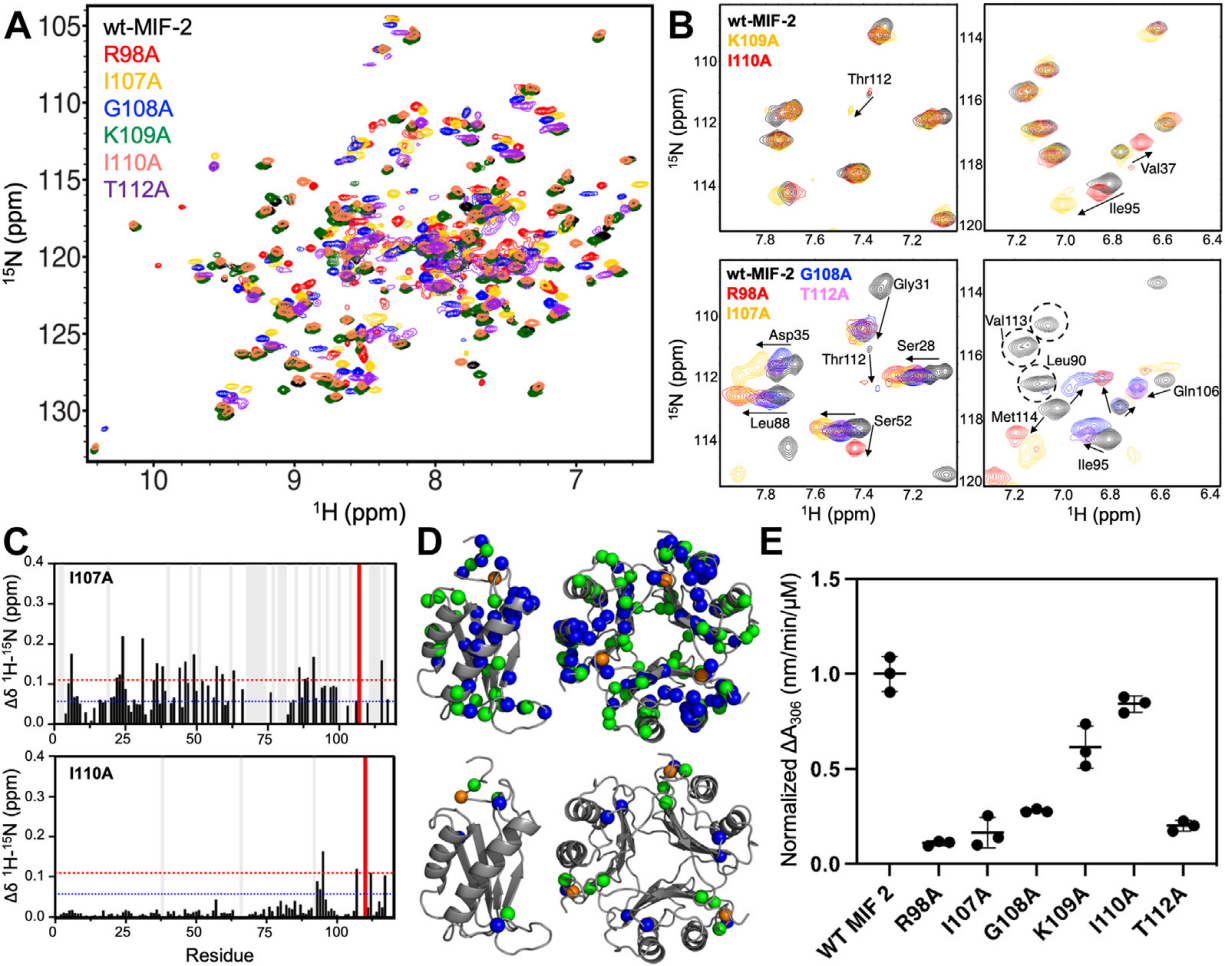
**Mutation of C-terminal residues modulatesMIF-2 structure and catalytic function.***A*, ^1^H-^15^N HSQC NMR spectral overlays of wt-MIF-2 (*black*) and variants (*colored* according to the legend), demonstrating a similar overall chemical shift dispersion. *B*, portions of the NMR spectra comparing variants that closely resemble wt-MIF-2 structure, dynamics, and function (K109A, I110A, *upper panels*) and those that diverge from wt-MIF-2 structure, dynamics, and function (R98A, I107A, G108A, T112A, *lower panels*). Sites of significant chemical shift perturbation in these panels are denoted by *arrows* and wt-MIF-2 resonances that broaden beyond detection in the majority of variants are *circled*. *C*, combined ^1^H^15^N chemical shift perturbations (*black bars*) in MIF-2 caused by mutations. Representative MIF-2 variants I107A and I110A are shown. *Blue* and *red* dashed lines represent the 10% trimmed mean of all shifts and 1.5σ above the mean, respectively. *Gray vertical bars* denote sites of NMR line broadening and red bars indicate the mutation site. The NMR composite chemical shifts were determined by $\Delta \delta =\sqrt{\left({\Delta \delta }_{HN}^{2}+{\Delta \delta }_{NH}^{2}/25\right)/2}$. *D*, summary of NMR spectral perturbations caused by I107A (*upper*) and I110A (*lower*) mutations, where chemical shift perturbations (*green spheres*, defined as 1.5σ above the 10% trimmed mean of all shifts) and sites of line broadening (*blue spheres*; defined as a loss of ≥50% of wt-resonance intensity) are mapped onto the MIF-2 trimer. Sites of mutation are indicated by *orange spheres*. *E*, tautomerase assay monitoring an enol-borate complex of 4-hydroxyphenyl pyruvate (4-HPP, λ_max_ = 306 nm) produced by MIF-2. n = 3 to 4 in each case and data scatter displays error between assays. MIF-2, D-dopachrome tautomerase; NMR, nuclear magnetic resonance.

Mutation of these C-terminal residues divided the six variants into two resultant categories; those with wildtype (wt)-like enzymatic activity and those with strongly attenuated activity, where the latter demonstrate allosteric reciprocity to the N terminus. The keto-enol tautomerase function of the MIF superfamily is well-studied and presents a straightforward functional handle to assess the impact of structural perturbation on MIF-2 *in vitro*. Variants K109A and I110A comprise the “wt-like” group, retaining 60% and 83% of wt-MIF-2 enzymatic activity, respectively. Variants R98A, I107A, G108A, and T112A fall into the latter group, having at most ∼30% of the activity of wt-MIF-2 (Fig. 2). Though the thermal stabilities of two variants, R98A and I107A, are below those of wt-MF-2 and the other variants, all denaturation profiles are of similar shape and the secondary structures of all MIF-2 variants are consistent (Fig. S1), suggesting no significant structural decomposition from the mutations. Due to the number of variants used in this study, we discuss in detail a single representative MIF-2 variant of each category going forward: I110A and I107A of the wt-like and non-wt-like groups, respectively.

### NMR spectral perturbations negatively correlate with enzymatic activity and suggest allosteric coupling

To investigate whether the C-terminal residues of MIF-2 sustain the allosteric relay from Phe100 and explain the variation in enzymatic activity, we used solution NMR to determine changes in local structure, dynamics, and inter-residue communication. ^1^H^15^N TROSY-HSQC NMR spectra of wt-MIF-2 compared to C-terminal variants (Fig. 2) revealed a negative correlation between enzymatic activity and the degree of structural and dynamic perturbation in MIF-2, in agreement with prior reports (8, 9). The I107A variant perturbed regions distal to the mutation site *via* chemical shift perturbations and the greatest extent of line broadening of any variant, indicative of heightened dynamics across the entirety of the protein and supporting the involvement of Ile107 in intramolecular communication. The observed spectral perturbations correlate very well with regions of allosteric functional significance from our prior studies of MIF and MIF-2 (8, 9, 11), including monomer interfaces in X-ray crystal structures (β2: residues 38–42, β3: 46–49, β6,7: 105–112) as well as residues involved in hydrophobic interactions between the core β-sheets and α-helices (Leu3, Leu22, Ile59, Phe80, Phe81, Fig. 2). Other affected sites include N-terminal Phe2 and Leu3, which along with the global alterations caused by the I107A mutation, likely drive the 82% decrease in enzymatic activity from wt-MIF-2. Other variants with strongly attenuated catalytic activity—R98A, G108A, and T112A—exhibit similar responses to mutation, namely widespread chemical shift perturbations and line broadening. R98A MIF-2, the most structurally perturbative C-terminal variant of those tested (based on NMR chemical shifts), is most affected at the hydrophobic core (Leu3, Leu18, Leu22, Ala26, Val37, Val39, Val41, Ile61, Phe80, Phe81, Phe83, and Ile95). G108A and T112A MIF-2 variants each have fewer hydrophobic core perturbations, possibly due to the more solvent exposed positions of those residues at the C terminus. The total number of chemical shift perturbations and line broadened resonances follows a trend of R98A > I107A > T112A > G108A > K109A > I110A that directly correlates with the percent decrease in enzymatic activity (Figs. 2; S2 and S3).

In contrast, the I110A mutation causes modest structural and dynamic effects on MIF-2. Chemical shift perturbations and line broadening occur only at sites surrounding the mutation, at flexible loop regions, and at the ends of β-strands (Fig. 2). Remarkably, the affected residues include other “allosteric pathway” connections, such as Ile107 and Thr112 that lie at the monomer–monomer interface, several of which are at least 7 Å away from Ile110, demonstrating that relatively small structural effects can be sensed by functionally coupled distal sites. It is worth noting that the side chain of Ile110 points outward into the solvent and the replacement of the bulky Ile side chain with an alanine methyl group should not affect the neighboring amino acids as strongly, explaining the smaller structural perturbations and near-wt enzymatic activity. Mutation of Lys109 perturbs the structure more strongly than Ile110 when examining NMR chemical shifts, though most still cluster around the mutation site (Fig. S2) and both variants induce similarly low levels of NMR line broadening. The stronger effect of K109A on the local chemical environment may then be due to the side chain orientation of Lys109, which points toward the monomer interface, in contrast to Ile110.

### NMR spin relaxation highlights an inverse relationship between MIF-2 structural flexibility and catalytic function

Conformational fluctuations on multiple timescales can affect protein function and influence ligand binding events (12, 13, 14). These include ps-ns bond vector motions that organize ligand binding sites and routes of chemical signaling or report on global tumbling, as well as higher energy barrier μs-ms processes that affect folding and catalytic mechanisms (15). To determine how (or if) altered structural dynamics of MIF-2 variants contribute to the measured catalytic activities, we used *T*_1_, *T*_2_, and heteronuclear ^1^H-[^15^N] NOE spin relaxation experiments. *T*_1_ and *T*_2_ values for wt-MIF-2 and MIF-2 variants suggest similar rotational correlation times (τ_c_) and structural compactness. The average *T*_1_ and *T*_2_ across all residues of wt-MIF-2 were 1154 ± 57 ms and 51 ± 5.4 ms, respectively, in line with values expected of a compact ∼35 kDa protein at 600 MHz and 30 °C and consistent with a τ_c_ ≈ 20 ns (16). Given that MIF-2 is a trimer of 37 kDa, its behavior likely differs from a globular monomeric protein, accounting for slight variations in the expected *T*_1_, *T*_2_, and τ_c_ for a monomer of similar molecular weight. The average *T*_1_ and *T*_2_ values across all MIF-2 variants were determined to be nearly identical to those of wt-MIF-2, at 1199 ± 64 ms and 51 ± 1.2 ms, respectively. Thus, despite local fluctuations in per-residue plots of *T*_1_/*T*_2_ (Fig. S4), especially for variants with dramatically attenuated catalytic function, overall molecular tumbling rates are unaffected by the mutations.

**Figure 3:**
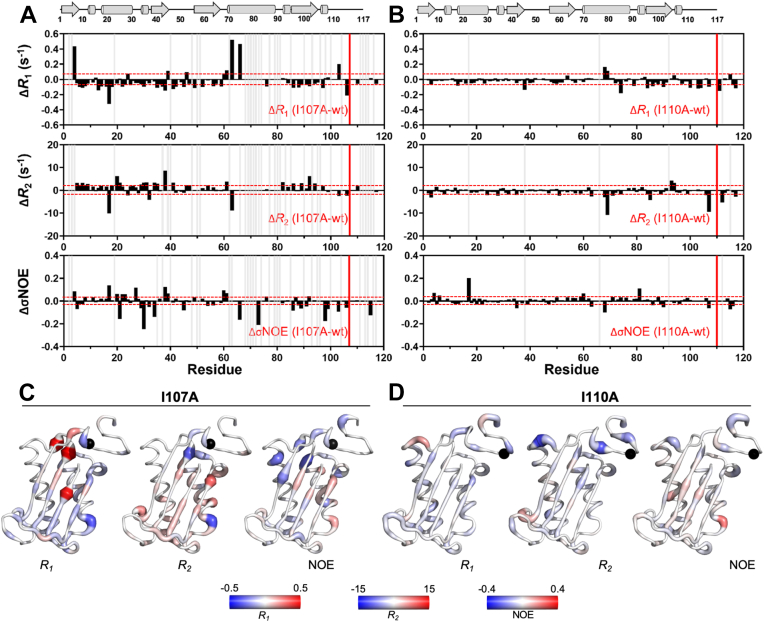
**Per-residue differences in *R***_**1**_**, *R***_**2**_**, and**^**1**^**H-[**^**15**^**N] NOE relaxation parameters for MIF-2 variants.***A*, relaxation parameters plotted for I107A (*R*_1_, *R*_2_, or NOE)_MUT_ – (*R*_1_, *R*_2_, or NOE)_WT_. *B*, identical parameters plotted for I110A MIF-2 as (mutant) – wt). In both panels, positive values of Δ*R*_1_ or Δ*R*_2_ indicate greater flexibility in the mutant, while negative values indicate greater flexibility in wt-MIF-2. In contrast, negative Δ_NOE_ values indicate a more flexible mutant, while positive values indicate a more flexible wt-MIF-2. *Cartoons* at top show the secondary structure elements of MIF-2 (not to scale). *Horizontal dashed red lines* indicate ± 1.5σ from the 10% trimmed mean of all *R*_1_, *R*_2_, and NOE datasets, respectively. Relaxation parameters that failed to reach statistical significance were defined as 0.04 ≥ Δ*R*_1_ ≤ −0.09, 2.04 ≥ Δ*R*_2_ ≤ −1.63, and 0.03 ≥ ΔNOE ≤ −0.04. *C* and *D*, per-residue heat maps of differences in *R*_*1*_, *R*_*2*_, and ^1^H-[^15^N] NOE relaxation parameters for (*C*) I107A and (*D*) I110A displayed on the MIF-2 monomer. *Blue* and *red colors* indicate a more negative or positive value, respectively. *Colored bars* indicate the scale of the change based on the color intensity. MIF-2, D-dopachrome tautomerase.

Representative *R*_1_ and *R*_2_ relaxation rates are shown as difference plots (mutant – wt) to quantify how the variants diverge from wt-MIF-2 on a single amino acid basis (Fig. 3, Tables S1–S6 and Figs. S5–S7). Unsurprisingly, I110A does not strongly perturb the dynamics of wt-MIF-2. A modest, but global, decrease in *R*_1_ and *R*_2_ is observed, with the greatest effect in the latter third of the protein (residues 70–117). In this region, the average *R*_1_ has over a 2-fold difference compared to the average *R*_1_ of residues 1 to 60. The effect on *R*_2_ is modest, except for decreases in residues Thr69 and allosteric pathway residue Ile107 (Fig. 3). Changes in relaxation parameters are small in I107A MIF-2 as well, but they appear in a different pattern. The *R*_1_ values of most residues are moderately decreased, but unlike I110A, these effects generally occur consistently across the whole protein. This effect is particularly pronounced in residues 1 to 60, where I110A has minimal effect. Some residues with noticeable elevations in *R*_1_ are Glu4, Ser63, and Val66, suggesting only small enhancements of fast timescale fluctuations.

Residues with elevated *R*_*1*_ parameters lie closest to Ile107 of the same monomer. An Ile107 residue from an adjacent monomer would be located on the other side of the β-sheet structure, far from the perturbed sites. Upon mutation to alanine, the Ala107 side chain is unlikely to directly interact with residues displaying elevated *R*_*1*_, especially since both *R*_*1*_ and *R*_*2*_ parameters hint at a more rigid structure around the site of mutation, relative to wt-MIF-2 (Fig. 3). Ala107 from the adjacent monomer may transmit dynamic perturbations to Pro1 through the allosteric network, which then propagates to Ser63 and Val66. Though numerous MIF-2 residues are not observed in relaxation studies of I107A due to extensive line broadening, many sites with quantifiable changes in relaxation parameters are in close proximity to Ser63 and Val66 and are directly next to Glu4, in the middle of a β-strand at the enzymatic site. *R*_2_ values of the I107A variant are mostly elevated, in contrast to the global depression caused by I110A (Fig. 3) and consistent with prior findings that enhanced protein motion contributes to diminished enzymatic activities of MIF and MIF-2. Local changes in relaxation rates are more clearly visualized in heat maps of MIF-2 variants, where I107A has a larger number and magnitude of quantified change across the protein, particularly at the N terminus (rightmost β-sheets and α-helix) when compared to I110A (Fig. 3).

Other C-terminal variants that strongly attenuate MIF-2 enzymatic activity (R98A, G108A, T112A) have global effects on *R*_*1*_ and *R*_*2*_, similarly to I107A (Figs. S5 and S6). The lack of an obvious pattern in relaxation difference plots when comparing these variants to wt-MIF-2 suggests that each mutated residue has a unique contribution to MIF-2 dynamics. Generally, heightened flexibility of the C-terminal variants is a diagnostic of poor enzymatic function, and one observable trend is that *R*_*1*_ gradually decreases following the sequence R98A, I107A, G108A, K109A, I110A, and T112A, culminating in a near protein-wide depression of *R*_*1*_ for T112A. This inversely follows the trend of enzymatic activity, which increases in the same order (Fig. 2). When visualized on heat maps of Δ*R*_*1*_, the core β-sheet structure in MIF-2 is quite red (positive values) for R98A and trends toward increasingly blue shades (negative values) in sequential order of mutation to T112A (Fig. S8). These data suggest that the location of the amino acid at the C terminus and its unique effect on the MIF-2 allosteric network has site-specific influence over fast timescale dynamics that contribute to proper MIF-2 function (9).

The ^1^H-[^15^N] NOE (σ_NOE_) was also used to evaluate fluctuations of the MIF-2 backbone and revealed a consistent pattern in the variants. Despite the fact that all MIF-2 variants maintain an average σ_NOE_ that is similar to wt-MIF-2 when considering the entire structure, per-residue σ_NOE_ values more tightly cluster around those of wt-MIF-2 in variants with wt-like activity, as seen in K109A and I110A. In contrast, I107A has a noticeably larger σ_NOE_ range, with many negative Δ_NOE_ across the protein, suggesting heightened flexibility in this variant (Figs. 3, S9 and S10). Other variants with depressed catalytic activity and high flexibility *via* widespread negative Δ_NOE_ values (R98A, I107A, G108A, and T112A) display heat maps with greater blue density (Fig. S8). These data echo the pattern observed in analysis of NMR chemical shift perturbations and line broadening, namely that variants with greater conformational plasticity on fast-to-intermediate timescales most strongly attenuate MIF-2 enzymatic activity.

The per-residue variance of σ_NOE_ and overall flexibilities of the I107A and I110A case studies are echoed in order parameters (*S*^2^) determined from model-free analysis of multifield relaxation measurements (Fig. S11). Although line broadening caused by the I107A mutation precluded model-free analysis of nearly 30 resonances, plots of *S*^2^ and Δ*S*^2^ reveal fluctuations in backbone bond vectors (*i.e.*, large negative Δ*S*^2^) in residues 20 to 30, which show highly depressed σ_NOE_, substantial structural fluctuations in MD simulations (*vide infra*), and are implicated in ligand binding. Also affected are residues 60 to 80, which contain hotspots of mutation-induced allosteric crosstalk determined by MD simulations and are bracketed by large clusters of exchange broadening. In contrast, *S*^2^ values of I110A are almost identical to those of wt-MIF-2, consistent with relaxation parameters discussed earlier.

### Mutation-induced structural changes to the C terminus alter allosteric communication in MIF-2

**Figure 4:**
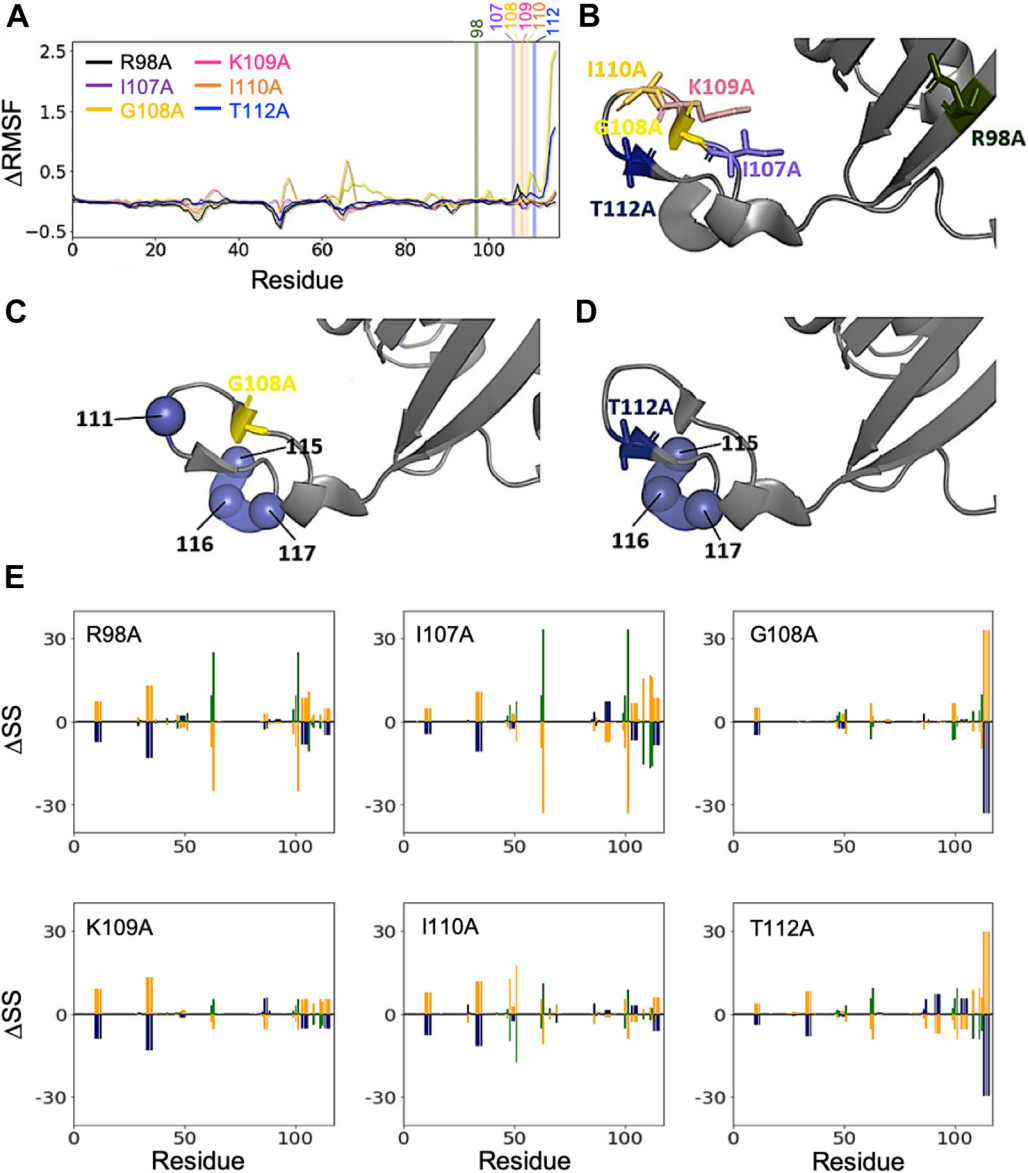
**Structural and dynamic effects of MIF-2 variants*****in silico.**A*, change in RMSF between wt-MIF-2 and R98A (*green*), I107A (*purple*), G108A (*yellow*), K109A (*red*), I110A (*orange*), and T112A (*blue*) variants. Bars indicate mutation sites and are colored according to the legend. *B*, MIF-2 (*gray* cartoon) with mutated residues shown as licorice and colored according to (*A*). *C*, the G108A variant and residues with ΔRMSF at least 2σ above the mean shown as *blue spheres*. *D*, analogous to (C) but for the T112A variant. *E*, change in secondary structure due to R98A, I107A, G108A, K109A, I110A, and T112A mutations, calculated as probability of secondary structure (mutant—wt-MIF-2) throughout sets of MD simulations. Negative values indicate the loss of a secondary structure element, and positive values indicate the adoption of a secondary structure element upon mutation. Secondary structure elements are colored as *blue* (helix), *green* (sheet), and *orange* (coil). MD, molecular dynamics; MIF, migration inhibitory factor; RMSF, root-mean-squared fluctuation.

We analyzed the change in mutation-induced flexibility by computing the change in root-mean-squared fluctuation (ΔRMSF) between wt-MIF-2 and the C-terminal variants (Fig. 4). Consistent with NMR data, we find that low-activity variants, most notably G108A and T112A, increase mobility of the C terminus significantly, which propagates to residues 28 to 31, 33 to 36, 50 to 54, and 65 to 68 *via* more moderate gains in flexibility that likely hinder catalytic activation. To further understand the mutation-induced structural changes to MIF-2, we computed the per-residue secondary structure and observed the six MIF-2 variants to be similar. MIF-2 variants, in general, display modest secondary structure fluctuations across the protein sequence, consistent with a stable symmetric trimer. However, there is a prominent helix-to-coil conversion of the C terminus due to the G108A and T112A mutations, commensurate with the increased RMSF and heightened ps-ms dynamics observed by NMR spin relaxation and line broadening (Fig. 4).

**Figure 5:**
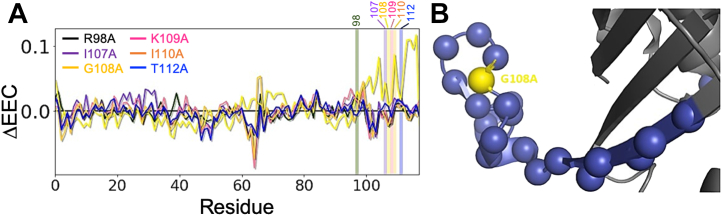
**Mutation-induced changes in MIF-2 allosteric communication.***A*, change in electrostatic eigenvector centrality (ΔEEC) upon R98A, I107A, G108A, K109A, I110A, and T112A mutations. Stacked ΔEEC plots for each individual MIF-2 variant is shown in Fig. S11. *B*, protein representation of MIF-2 (*gray cartoon*) with Ala108 colored *yellow*. The residues with an increase in EEC due to the G108A mutation at least 2σ above the mean are shown as *blue spheres*.

We gained insight into MIF-2 allosteric communication *via* an electrostatic eigenvector centrality (EEC) metric by computing the correlation of electrostatic energy (calculated from the Kabsch–Sander formalism) between residues throughout an MD trajectory, allowing us to pinpoint those residues most important to the electrostatic network. By taking the difference between wt-MIF-2 and the variants, we identified residues whose importance to the network changes significantly upon mutation (Figs. 5 and S12). EEC has been shown to correlate with NMR chemical shifts and therefore is complementary to our experiments aimed at understanding the dynamics of regions that experience significant changes in their chemical environment due to a perturbation such as a point mutation. Again taking G108A MIF-2 as an example, this mutation induces a strong increase in the C-terminal ΔEEC, suggesting it becomes more strongly coupled to the MIF-2 allosteric network, commensurate with both the mutation-induced increase in RMSF as well as the calculated helix-to-coil conversion measured for this region of the protein. While the C-terminal effect is not as strong for other variants, substantial differences are observed near residues 60 to 70, a region that was shown to be flexible by NMR order parameters and ^1^H-[^15^N] NOEs and critical for allosteric crosstalk in an earlier study of MIF-2 (9).

### Neutrophil recruitment is differentially affected by C-terminal mutations, in vivo

**Figure 6:**
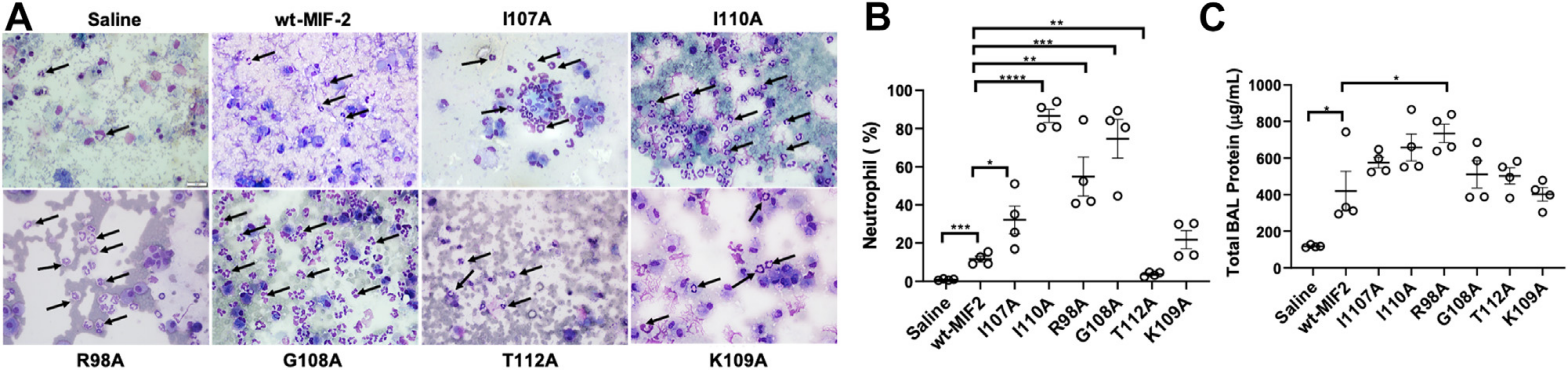
**C-terminal allosteric mutations modulate CD74-mediated inflammatory activity.***A*, BAL cytosmears showing neutrophil (*arrows*) recruitment by MIF-2 variants. Saline and wt-MIF-2 are shown as controls. *B*, MIF-2 variants enhance neutrophil recruitment in murine BAL fluid *in vivo* to varying degrees. *C*, MIF-2 variants generally enhance BAL fluid protein levels, which is a surrogate marker for alveolar-capillary leak and pulmonary edema *in vivo*. Data are expressed as mean ± SEM (n = 4 for each group). N = 4, ∗*p* < 0.05; ∗∗*p* < 0.005; ∗∗∗*p* < 0.001; ∗∗∗∗*p* < 0.0001; BAL, bronchoalveolar lavage; CD74, cluster of differentiation 74; MIF, migration inhibitory factor; Scale bar: 100 μm.

Through molecular dynamics simulations, the C-terminal region of MIF has been previously demonstrated to be critical for CD74 receptor activity (17). Mutations in the MIF C terminus resulted in diminished neutrophil recruitment in murine lungs *in vivo* (6, 10), which is the established assay for CD74 activation by MIF proteins (18). However, the functional significance of comparable MIF-2 mutations on murine lung inflammation was unknown. To test this effect, saline control, wt-MIF-2, and C-terminal variants of MIF-2 (R98A, I107A, G108A, K109A, and T112A) were delivered intratracheally into murine lungs, and the percentage of neutrophils and total protein (a marker for pulmonary edema) were measured from the bronchoalveolar lavage (BAL) fluid (Fig. 6).

Mutation of MIF-2 C-terminal residues affected neutrophil recruitment in a manner that suggests Arg98, Ile107, Gly108, Lys109, and Ile110 may not be involved in binding and stabilization of CD74 but are instead modulators of CD74 receptor activity (Fig. 6*A*). This speculation is driven primarily by the observation that these variants *increase* neutrophil recruitment, where amino acids comprising the direct binding site for CD74 would be more likely to strongly attenuate neutrophil recruitment if mutated. Interestingly, neutrophil recruitment is only diminished by a T112A mutation. To further rationalize the functional consequences of the MIF-2 variants and connect to allosteric signaling and C-terminal flexibility, we used the previously described EEC to assess the ability of the C-terminal residues to propagate electrostatic couplings throughout the protein, serving as a proxy for allosteric communication. The clearest example for a biophysical rationale appears when focusing on the representative G108A and T112A variants, which have opposite impacts on MIF-2 signaling *in vivo*. Changes in flexibility and secondary structure show that the C terminus becomes significantly more flexible upon mutation of G108 and T112, explained by a secondary-structure conversion from helix to coil (Figs. 4 and 6). Interestingly, the G108A variant shows significantly increased EEC and therefore a much greater ability to propagate allosteric information throughout the protein (*i.e*., stronger allosteric coupling), suggesting that the diminished neutrophil recruitment observed for the T112A variant may be due to an increase in flexibility caused by the helix-to-coil secondary structure transition, without the compensatory increase in EEC, as seen for G108A. We therefore speculate that a distinct C-terminal conformation is necessary for neutrophil recruitment, assuming no significant increase in allosteric communication. This finding is supported by a concurrent study which revealed unexpected mobility of the MIF-2 C terminus resulting from ion or ligand binding, phosphorylation, or protein–protein interactions. All variants of MIF-2 increase the total BAL protein levels, with R98A showing the most significant increase compared to wt-MIF-2. The increase in total BAL protein, a marker for pulmonary edema, coincides well with increased neutrophil recruitment by all variants except for T1112A (Fig. 6). While neutrophils are known to contribute to increased lung permeability directly by disrupting epithelial cell junctions, increased alveolar-capillary permeability can occur in the absence of neutrophil recruitment (19, 20), which provides a biophysical rationale for the response of T112A MIF-2. In light of prior *in vivo* studies of MIF-2 indicating that N-terminal and solvent channel mutations suppressed CD74-induced neutrophil recruitment (9), allosteric control of the CD74 binding event now appears to involve C-terminal residues beyond those studied here, since only one mutation, T112A, suppresses CD74 activation.

## Discussion

The C-terminal residues of MIF-2 studied in this work are distant from the catalytic N-terminal Pro1. However, as MIF-2 is a homotrimer, the N and C termini of adjacent monomers actually appear closer in space. The C-terminal residues closest to the catalytic site are Arg98 and Ile107, which are ∼4 Å away from both the aromatic ring of Phe2 and from Ser62 (Fig. S13), the residue that mediates communication between Pro1 and Phe100 at the previously reported solvent channel allosteric site. At these distances, it may be possible for Arg98 or Ile107 to interact with Phe2, which in turn could affect the catalytic base, Pro1 and destabilize the protein, which is supported by altered thermal stabilities of the R98A and I107A variants (Fig. S1). A disruption of enzymatic activity through perturbation of the allosteric Ser62 alone is also unlikely since prior studies showed only modest changes in enzymatic activity with mutations at Ser62 (9), while the R98A mutation decreases catalysis by 89%.

The MIF residue analogous to Arg98 of MIF-2 is Asn97, which has been shown to directly interact with the 4-hydroxyphenyl pyruvate (4-HPP) pseudosubstrate in a crystal structure (21). There is no X-ray crystal structure of MIF-2 bound to 4-HPP; however, ^1^H^15^N HSQC NMR titrations of 4-HPP into wt-MIF-2 do not demonstrate strong chemical shifts at Arg98, suggesting that this residue does not strongly interact with 4-HPP in MIF-2 (Fig. S14). In fact, NMR data suggest the overall interaction point(s) of 4-HPP with MIF-2 may be quite different from MIF. The 4-HPP binding residues within MIF, determined crystallographically and biochemically, show essentially no chemical shift perturbations in 4-HPP titrations of MIF-2. Rather, strong perturbations and line broadening are observed between residues 60 and 80, an allosteric hotspot in MD simulations, and at the C terminus. While this site is close in three-dimensional space to the 4-HPP binding site of MIF, it is clearly unique. The fact that the MIF and MIF-2 active site architectures and charges differ considerably despite conservation of a global fold (22) and MIF-2 tautomerizes 4-HPP with 10-fold lower activity than MIF (1) also suggests that MIF family proteins may have different interactions with 4-HPP. The molecular underpinnings of the MIF-2-4-HPP complex, as well as complexes with other pseudosubstrates and inhibitors, are currently being investigated by our group for new comparisons between MIF and MIF-2.

In ^1^H^15^N TROSY-HSQC NMR spectral overlays, R98A and I107A variants notably perturbed the MIF-2 hydrophobic core, correlating with weaker enzymatic activity and structural instability (Figs. 2 and S1). Interestingly, variants G108A and T112A also have substantially diminished catalytic activity, yet do not disrupt the hydrophobic network to nearly the same extent, which may be due to R98A and I107A being in closer proximity to the core β-sheet of MIF-2. Mutation of Gly108 to alanine actually increases the thermal stability of MIF-2 by nearly 4 ^°^C, and this stabilizing effect points to the importance of the MIF-2 hydrophobic β-sheet core for its structure. The impact of Gly108 on residues outside of the hydrophobic core, particularly those at the C terminus, must account for the large decrease in catalytic activity. A similar effect is observed in the T112A variant, and the >7 Å distance of Gly108 and Thr112 from all MIF-2 functional sites strongly support long-range chemical signaling as a means of modulating MIF-2 activity.

Residues previously identified in the allosteric networks affecting MIF and MIF-2 catalysis, other than Pro1, were not conserved but had similar chemical properties. Mutations in the N-terminal portion of the allosteric pathways yielded structures and activities very similar to, or very divergent from, wt-MIF and MIF-2. Interestingly, mutagenesis of the most chemically divergent C-terminal amino acids in MIF and MIF-2 (*i.e.*, Trp108/Lys109 and Asn109/Ile110 in MIF/MIF-2) yielded the most wt-like structure-function signatures, while identical or highly similar C-terminal residues in MIF and MIF-2 (*i.e.*, Asn97/Arg98, Val106/Ile107, Gly107/Gly108, and Ser111/Thr112) caused drastic effects when mutated. Each mutation target in MIF-2 is conserved among other species, with the exception of the Phe100 allosteric site (9) (a somewhat common feature of protein allostery) and Ile110. Though Lys109 and Ile110 are located on the surface of the MIF-2 structure with their side chains exposed to the solvent, they appear at the monomer–monomer interface along with Thr112, which may be the driving force for any functional effects observed in the absence of large structural or dynamic perturbations.

The C-terminal mutations explored here caused perturbations at the C terminus itself, indicating a connection between these individual amino acids. Further, in support of the MIF-2 C terminus communicating with allosteric residues that were previously explored (9), mutation of C terminal amino acids structurally perturbed Phe2, Ser62, and Phe100 of the enzymatic and solvent cavity allosteric sites, respectively. Thus, mutations in the N- and C-terminal regions of MIF-2 yielded reciprocal structural effects and conformational fluctuations *via* NMR. Though these structural studies already support our hypothesis of conserved allostery in the MIF superfamily, varying degrees of NMR line broadening in the spectral overlays of MIF-2 C-terminal variants suggested substantial changes in the flexibility of the system and NMR spin relaxation experiments explored whether the loss or preservation of structure and activity could be explained by underlying dynamic signatures.

Several MIF-2 variants, namely K109A and I110A, do not experience marked structural change upon mutation and therefore closely resemble the wt-MIF-2 HSQC fingerprint (Figs. 2 and 3). However, these variants display stronger motional perturbations in the region of residues 70 to 117 which includes α2, β5, and the C-terminal tail of MIF-2, which has important implications for monomer–monomer contact as well as CD74 activation. The lack of flexibility in the β-strand rich 1 to 60 region may explain the wt-like nature of the K109A and I110A variants (Fig. S7). In contrast, non-wt-like *R*_1_ and *R*_2_ relaxation parameters observed in variants with highly attenuated enzymatic activity (R98A, I107A, G108A, and T112A) appear across the entirety of the protein, including α1 and β-strands 1 to 4 that participate in monomer–monomer contact and form most of the core β-sheet structure, which has been shown to transmit chemical signals for allostery (23). These variants also generally have larger and more negative ΔσNOE values, supporting a heightened flexibility. In contrast to prior studies of MIF and MIF-2 N termini, it is very difficult to extract a pattern for these C-terminal variants, relative to wt-MIF-2 or previously studied variants, that support protein dynamics as the sole driver of MIF-2 function. While ps-ns timescale flexibility in MIF proteins certainly contributes to subtle structural rearrangements that affect enzymatic activity, our results suggest that each amino acid affects allosteric crosstalk uniquely and in a complicated manner. This is not unusual when considering prior biophysical studies of MIF or MIF-2, which continue to prove these molecules to be much more complex than their size or structure would portend.

Based on its homology to MIF and the established connection between MIF enzymatic function and biological signaling (24), studies of the MIF-2 C terminus are also relevant to its interaction with CD74. Obtaining experimental structures of MIF proteins bound to CD74 has proven extremely challenging, possibly due to a requirement for trimerization of the extracellular portion of CD74. Indeed, part of the CD74 trimer structure has been determined by NMR (25), but neither the trimerization domain nor the entire extracellular domain interacted with MIF. A related study suggested the transmembrane domains of CD74 also contribute to its trimerization (26). More recently, a fusion of extracellular CD74 and MBP was shown to bind MIF at 10% of the capacity of full-length CD74, implying a required conformational change in the trimeric extracellular domain even in the presence of trimeric MBP, which mimics the transmembrane portion (27). Despite these obstacles, it is reported that the interaction stoichiometries of MIF and MIF-2 with CD74 may differ due to a single amino acid insert in the MIF-2 sequence (17). However, this difference does not manifest functionally, as both proteins bind CD74 with nanomolar affinity to activate ERK1/2 signaling to similar levels in macrophages (1). Therefore, differences in the topography of binding coupled to residue-specific transmission of allosteric signals are useful starting points for drug targeting strategies. In the absence of additional structural information, *in silico* docking and molecular simulations of the interaction have driven new hypotheses. MIF and MIF-2 are proposed to bind CD74 with a similar “hydrophobic cradle” at the C terminus, along with a salt bridge at Glu103 and an interaction at the disordered loop comprised of residues 63 to 69 (17). We find that the MIF-2 C terminus (specifically residues 112–116) is highly perturbed by the mutations, most obviously through NMR chemical shifts and line broadening. Spin relaxation experiments also identified Glu103 to routinely display altered fast timescale motions. Residues 63 to 69 were similarly affected in both HSQC fingerprints and spin relaxation experiments as well as ΔEEC *in silico*, demonstrating the range of dynamic consequences of mutating residues in the allosteric network.

The amino acids investigated here (R98, I107, G108, K109, I110, and T112) were thought to comprise the CD74 activation site based on sequential and structural homology to MIF. The effects of these mutations on the MIF-2 N terminus and enzymatic activity, as well as the agreement of our data with the MIF-2 CD74 model (17), support these residues as CD74 modulators within the MIF-2 allosteric pathway. However, *in vivo* functional assays show that most of these variants actually enhance CD74 activation, the opposite of what one would expect from perturbations to the binding region. In comparison to MIF, mutation of the analogous residues (N97, G107, W108, N109, and S111) to alanine all decrease CD74 activation by at least 50% in neutrophil recruitment assays when compared to wt-MIF (6, 10). However, the C terminus of MIF-2 is two amino acids longer than MIF and is not well conserved between the two homologs. These differences in primary sequence and topographical binding to the CD74 receptor may account for the discrepancy between the MIF and MIF-2 CD74 activity *in vivo*. These studies further illuminate the functional subtleties dictated by sequence in the MIF superfamily, which are hidden behind their conserved architecture. Thus, future work will focus on other residues proximal to the C terminus to assess whether nearby sites attenuate CD74 binding and activation *in vivo*.

## Experimental procedures

### Protein expression and purification

Wildtype and mutant MIF-2 proteins were expressed and purified as previously described (4). MIF-2 was transformed into *E. coli* BL21-Gold (DE3) cells and grown in Luria Broth supplemented with 0.100 mg/ml ampicillin at 37 °C to an *A*_600_ of 0.6 to 0.8. Protein expression was induced at a final concentration of 1 mM isopropyl β-D-1-thiogalactopyranoside, and cells were incubated for an additional 18 h at 20 °C. Cells were then harvested by centrifugation and resuspended in a buffer of 20 mM Tris, 10 mM NaCl at pH 8.5 and supplemented with 1 mM PMSF. Cells were lysed on ice by ultrasonication, the lysate was clarified by centrifugation at 4 °C, and the supernatant was filtered through a 0.22 μm filter. The supernatant was loaded onto a Q-Sepharose anion-exchange column and washed with a buffer of 20 mM Tris, 10 mM NaCl at pH 8.5. MIF-2 was eluted using a 5% gradient of 20 mM Tris, 1 M NaCl at pH 8.5. MIF-2 was further purified with a 26/60 Superdex 75 column equilibrated with a buffer of 20 mM Tris, 150 mM NaCl at pH 7.4 to ∼95% purity (Fig. S15). Protein purity was determined by sodium dodecyl sulfate-polyacrylamide gel electrophoresis, and protein concentrations were determined by absorbance at 280 nm using ε_280_ = 5500 Μ^−1^ cm^−1^.

### Circular dichroism spectroscopy

Folding and thermal denaturation circular dichroism data were measured with a Jasco J-815 spectropolarimeter using a 0.2-cm quartz cuvette with 10 μm MIF-2 in a buffer of 20 mM sodium phosphate at pH 7.4. Thermal denaturation experiments were collected at 218 nm over a temperature range of 25 to 90 °C, sampling 1.5 °C at a rate of 1.5 °C/min.

### Enzymatic assays

For measurement of MIF-2 activity, a 100 mM solution of 4-HPP in 0.5 M ammonium acetate, pH 6.0, was prepared by overnight agitation at room temperature to generate its keto form. MIF-2 enzymatic activity was determined by monitoring the increase in absorbance at 306 nm caused by enol–borate complex formation between boric acid and 4-HPP in the reaction solution. Absorbance was first recorded with a mixture of 1.2 mM 4-HPP and 0.420 M boric acid, then the reaction was initiated by adding MIF-2 at a final concentration of 80 nM, and the final absorbance was recorded after incubation for 3.5 min. Data were analyzed according to previously published methods (3).

### NMR spectroscopy

NMR samples of MIF-2 were prepared as described earlier, but rather than Luria Broth media, MIF-2 expression was carried out in deuterated M9 minimal media containing MEM vitamins (Sigma-Aldrich) and ^15^NH_4_Cl and ^12^C-glucose as the sole nitrogen and carbon sources, respectively. Purified MIF-2 was dialyzed into a final NMR buffer of 20 mM Tris, 20 mM NaCl at pH 7.4 with 10% D_2_O and then concentrated to 0.5 to 1.0 mM. NMR experiments were performed on Bruker Avance NEO 600 MHz and Burker Avance III HD 850 MHz spectrometers at 30 °C. NMR data were processed using NMRPipe (28) and analyzed in Sparky (29) with the help of in-house scripts. Backbone assignments for wt-MIF-2 were obtained on ^2^H-^13^C-^15^N-labeled MIF-2 (prepared as described earlier) *via* TROSY HSQC (30), HNCA, HNCACB, HNCOCA, HNCO, and HNCOCACB experiments (31, 32) and deposited in the Biological Magnetic Resonance Data Bank (entry 50,790). Resonance assignments were able to be transferred onto the HSQC spectrum of each variant, and NMR chemical shift perturbations were quantified using the method of Bax *et al.* (33).

TROSY-based spin relaxation experiments were performed with the ^1^H and ^15^N carriers set to the water resonance and 120 PM, respectively. Longitudinal relaxation rates (*R*_*1*_) were measured with randomized *T*_1_ delays of 0, 20, 60, 100, 200, 600, 800, 1,200, 1,500, 2,000, and 2500 ms. These data were collected in a temperature-compensated manner with eight scans of 1024 and 256 points in the direct and indirect dimensions, respectively, over a 14 ppm ^1^H and 35 ppm ^15^N spectral range. Transverse relaxation rates (*R*_*2*_) were measured with randomized *T*_*2*_ delays of 0, 16.9, 33.9, 67.8, 136, 169, and 203 ms. These data were collected in a temperature-compensated manner with eight scans of 1024 and 256 points in the direct and indirect dimensions, respectively, over a 14 ppm ^1^H and 35 ppm ^15^N spectral width. The recycle delay in these experiments was 2.5 s (34). Longitudinal and transverse relaxation rates were extracted by nonlinear least squares fitting of the peak heights to a single exponential decay using in-house software. Uncertainties in these rates were determined from replicate spectra with duplicate relaxation delays of 20 (×2), 60, 200, 600 (×2), 800, and 1200 ms for *T*_1_ and 16.9, 33.9 (×2), 67.8, 136 (×2) and 203 ms for *T*_2_. The heteronuclear cross-relaxation rate (NOE) was obtained by interleaving pulse sequences with and without proton saturation and calculated from the ratio of peak heights from these experiments (34). These data were collected with a 5 s relaxation delay followed by a 3 s saturation (delay) for the saturated (unsaturated) experiments. Model-free analysis was carried out using the Lipari-Szabo formalism in RELAX with fully automated protocols (35).

### Titration of 4-HPP into MIF-2

^1^H-^15^N TROSY HSQC fingerprint spectra of 0.5 mM MIF-2 were collected at 30 °C in 20 mM Tris and 20 mM NaCl at pH 7.4 using the following ratios of protein-to-ligand (MIF-2:4-HPP)—1:0, 1:0.5, 1:1, 1:2, and 1:5. 4-HPP (Sigma-Aldrich) was dissolved in an identical NMR buffer of 20 mM Tris and 20 mM NaCl at pH 7.4 to a stock concentration of 15 mM. Saturation of MIF-2 with 4-HPP was followed until NMR chemical shift perturbations were no longer visible with subsequent additions of 4-HPP.

### MD simulations, RMSF, secondary structure, and EEC computational analyses

Six mutants of MIF-2 (R98A, I107A, G108A, K109A, I110A, and T112A) were created by introducing point mutations to all three monomers of wt-MIF (PDB 1KAN) (2), for a total of seven model systems. Each structure was solvated with a periodic water box containing sufficient counter ions to neutralize the systems. Simulations were run for 150 ns at 300 K using the Nanoscale MD (NAMD) software package (36) and the CHARMM36m force field (37), and the equilibrated final 50 ns were analyzed in the VMD software package (38).

Thermal fluctuations from MD simulations were used to describe flexibility *via* RMSF, change in secondary structure, and allosteric propagation of electrostatic information with the EEC formalism. The RMSF of a given residue j was calculated throughout an MD trajectory of N frames according to Equation 1:

([1]) $$RMSF\left(j\right)=\sqrt{\frac{1}{N}\sum_{i}^{N}{\left(x_{j\left(i\right)}-\left\langle x_{j}\right\rangle \right)}^{2}+{\left(y_{j\left(i\right)}-\left\langle y\right\rangle \right)}^{2}+{\left(z_{j\left(i\right)}-\left\langle z_{j}\right\rangle \right)}^{2}}$$

The difference in RMSF (ΔRMSF) was computed between MIF-2 variants to determine how the flexibility of the protein changed at the residue level due to single point mutations. A secondary structure element was assigned to each residue for each frame of the MD simulation according to the dictionary of secondary structure assignments (DSSP) function provided by mdtraj (39), where the possible simplified assignments are “helix”, “strand”, and “coil”. For every residue, a coefficient for each of the three assignments corresponded to the percent of the trajectory that a given residue is predicted to adopt the specified secondary structure assignment. The difference in secondary structure assignments was compared between MIF-2 variants to characterize how the secondary structure changed with different point mutations.

The EEC method describes allostery as the propagation of electrostatic information throughout a protein. Protein electrostatics were computed through the Kabsch–Sander formalism, where the interaction energy between each pair of residues is proportional to the coulombic coupling between the backbone hydrogen bond donor (amide) and acceptor (carbonyl) groups on adjacent residues, defined according to Equation 2:

([2]) $$E^{KS}=q_{1}q_{2}\left[\frac{1}{d_{ON}}+\frac{1}{d_{CH}}-\frac{1}{d_{CN}}-\frac{1}{d_{OH}}\right]f$$

where $q_{1}$ and $q_{2}$ represent the partial charges on the carbonyl carbon and amide hydrogen, approximated as 0.42*e* and 0.20*e*, respectively. The interatomic distance between atoms i and j was represented as $d_{ij}$ (in units of angstroms), and the dimensionality factor f was 332 kcal/mol Å, yielding an energy $E^{KS}$ with units of kcal/mol. Computing the energy matrices for each residue pair at each MD simulation frame resulted in a 3-dimensional matrix of size *N∗N∗*F, where *N* is the number of residues and *F* is the number of frames of the trajectory. Eigenvector centralities were then computed from this matrix according to the procedure developed in previous work (40). The calculated centrality of each residue characterizes its importance to the propagation of electrostatic information throughout the protein network. Moreover, the difference in EEC (ΔEEC) between MIF-2 variants describes how electrostatic information transfer is perturbed due to single point mutations.

### Animals

Wildtype adult male mice (10–12 weeks old) of genetic background strain (C57BL6/J) were purchased from Jackson Laboratory (Bar Harbor) and housed in a pathogen-free animal facility at Cooper University Hospital. Mice were administered a one-time intratracheal instillation of 50 μl of normal saline alone (vehicle) or 1 μg of either wt MIF-2 or one of the MIF-2 C-terminal allosteric variants (R98A, I107A, G108A, K109A, I110A, and T112A) resuspended in 50 μl normal saline. Intratracheal instillation was done following the methodology as previously described, with modifications^10^.The animal study protocol was approved by the Institutional Animal Care and Use Committee of Cooper University Hospital.

### Analysis of the BAL fluid

Following administration of different compounds, as mentioned above, the mice were sacrificed after 6 h to collect BAL fluid. Total cell counts in the BAL fluid were determined using the TC20 automated cell counter (Bio-Rad Laboratories), with the gating set at 6 to 12 μm. The total protein content in the BAL fluid was measured by the BCA assay (ThermoFisher Scientific). Approximately, 1 ml of the BAL fluid was pelleted at 1000 rpm for 10 min at 4 ^°^C; the pellet was resuspended in 200 μl 1X PBS and further cytocentrifuged at 1000 rpm for 10 min at RT for the pellet to be evenly spread on slides as a smear. The slides were air dried and stained with Hema III differential Quick stain (Fisher Scientific, Cat. No. 122-911). Neutrophils were counted manually in the smears following the methodology of Brauwer *et al.* (41) and Lorenzi *et al.* (42) to calculate their total percentage. Briefly, following staining, the cytosmear was randomly divided into 4 to 5 areas that housed the maximum number of neutrophils. Two hundred different inflammatory cell types (including neutrophils) were counted in these areas from which the percentage of neutrophils was calculated. At least four mice were used for the different groups of drugs instilled. Values are presented as means ± standard error of the mean (SEM). Statistical analyses for comparison of multiple groups were conducted using the one-way ANOVA or Student’s two-tailed *t* test when the assumptions of ANOVA were not met. *p* < 0.05 denoted statistical significance. All data were analyzed using GraphPad Prism statistical software.

## Data availability

Triple resonance NMR assignments for MIF-2 have been deposited in the Biological Magnetic Resonance Bank under ID 50790. All other data related to this work are available upon request to george_lisi@brown.edu or victor.batista@yale.edu.

## Supporting information

This article contains supporting information.

## Conflict of interest

The authors declare no conflicts of interest with the contents of this article.
