# Supplementary material for: Mapping N- to C-terminal allosteric coupling through disruption of a putative CD74 activation site in D-dopachrome tautomerase
Source: J Biol Chem. 2023 Apr 18;299(6):104729. doi: 10.1016/j.jbc.2023.104729 (PMC10208890; doi:10.1016/j.jbc.2023.104729)
Supplement: Supporting Tables S1–S6 and Figures S1–S15 [file mmc1.docx]

***Supporting Information***

**Mapping N- to C-terminal Allosteric Coupling through Disruption of a Putative CD74 Activation Site in D-dopachrome Tautomerase**

Emily Chen,^1,#^ Vinnie Widjaja,^1,#^ Gregory Kyro,^2^ Brandon Allen,^2^ Pragnya Das,^3^ Vineet Bhandari,^3^ Elias J. Lolis,^4^ Victor S. Batista,^2^* and George P. Lisi^1^*

^1^Department of Molecular Biology, Cell Biology, & Biochemistry, Brown University, Providence, RI 02903

^2^Department of Chemistry, Yale University, New Haven, CT 06511

^3^Section of Neonatology, Department of Pediatrics, Cooper University Hospital, Camden, NJ 08103

^4^Department of Pharmacology, Yale University School of Medicine, New Haven, CT 06511

#These authors contributed equally to this work

*Correspondence: george_lisi@brown.edu, victor.batista@yale.edu

**Table S1.** *R*_1_, *R*_2_, and ^1^H-[^15^N] NOE values for R98A MIF-2

**Table S2.** *R*_1_, *R*_2_, and ^1^H-[^15^N] NOE values for I107A MIF-2

**Table S3.** *R*_1_, *R*_2_, and ^1^H-[^15^N] NOE values for G108A MIF-2

**Table S4.** *R*_1_, *R*_2_, and ^1^H-[^15^N] NOE values for K109A MIF-2

**Table S5.** *R*_1_, *R*_2_, and ^1^H-[^15^N] NOE values for I110A MIF-2

**Table S6.** *R*_1_, *R*_2_, and ^1^H-[^15^N] NOE values for T112A MIF-2

**Figure S1.** Secondary structure and stability of MIF-2 C-terminal variants.

**Figure S2.** Combined ^1^H^15^N chemical shift perturbations of MIF-2 variants plotted and mapped onto structure

**Figure S3.** Normalized, concentration-corrected NMR resonance intensity changes caused by MIF-2 variants.

**Figure S4.** *T*_1_/*T*_2_ NMR parameters for wt-MIF-2 and variants

**Figure S5.** Summary of NMR *R*_1_ relaxation parameters.

**Figure S6.** Summary of NMR *R*_2_ relaxation parameters.

**Figure S7.** Regions of *R_1_* and *R_2_* dynamic perturbation in MIF-2 variants.

**Figure S8.** Heat map of per-residue differences in *R_1_*, *R_2_*, and ^1^H-[^15^N] NOE relaxation parameters

**Figure S9.** Summary of NMR ^1^H-[^15^N] heteronuclear NOE relaxation parameters.

**Figure S10.** Per-residue differences in *R*_1_, *R*_2_, and ^1^H-[^15^] NOE relaxation parameters for MIF-2 variants.

**Figure S11.** NMR order parameters for wt-, I107A and I110A MIF-2

**Figure S12.** Electrostatic Eigenvector Centrality plots for MIF-2 variants.

**Figure S13.** Potential interactions of the N-terminal and C-terminal allosteric residues.

**Figure S14.** 4-Hydroxyphenylpruvate titration into wt-MIF-2.

**Figure S15.** Size exclusion chromatograms for wt-MIF-2 and MIF-2 variants

**Table S1.** *R*_1_, *R*_2_, and ^1^H-[^15^N] NOE values for R98A MIF-2

**Table S2.** *R*_1_, *R*_2_, and ^1^H-[^15^N] NOE values for I107A MIF-2

**Table S3.** *R*_1_, *R*_2_, and ^1^H-[^15^N] NOE values for G108A MIF-2

**Table S4.** *R*_1_, *R*_2_, and ^1^H-[^15^N] NOE values for K109A MIF-2

**Table S5.** *R*_1_, *R*_2_, and ^1^H-[^15^N] NOE values for I110A MIF-2

**Table S6.** *R*_1_, *R*_2_, and ^1^H-[^15^N] NOE values for T112A MIF-2

**Figure S1.** Secondary structure and stability of MIF-2 C-terminal variants. **(A)** Far-UV circular dichroism spectroscopy showing the α-β secondary structure is retained upon mutation of allosteric residues **(B)** Assessment of thermal stability of MIF variants (λ = 218 nm) showing that thermal stabilities are generally similar upon mutation of allosteric residues, with the exception of R98A (red) and I107A (orange). Solid lines represent best fits of the data. Thermal stabilities indicated in parentheses.

**Figure S2. (A)** Combined ^1^H^15^N chemical shift perturbations (black bars) in MIF-2 caused by mutations. Blue and red dashed lines represent the 10% trimmed mean and 1.5σ above the mean, respectively. Gray bars denote regions of NMR line broadening and red **bars indicate the mutation site. (B)** Summary of NMR spectral perturbations caused by mutations in MIF-2, where chemical shift perturbations (green spheres, defined as 1.5σ above the 10% trimmed mean of all shifts, as in **Figure 1B**) and sites of line broadening (blue spheres; defined as a loss of ≥50% of wt- resonance intensity) are mapped onto the MIF-2 monomer and trimer. Sites of mutation are indicated by orange spheres.

**R98A**

**I107A**

**G108A**

**K109A**

**I110A**

**T112A**

**Figure S3.** Normalized, concentration-corrected NMR resonance intensity changes caused by MIF-2 variants. wt-MIF-2 and MIF-2 variant peak intensities were recorded with Sparky’s Peak Height Analysis tool. Peak intensities of MIF-2 variants were corrected for concentration by Intensity_(mut)_ / Intensity_(wt)_ based on known protein concentrations. These values were then divided by the peak intensities of wt-MIF-2 and normalized against the maximum intensity value for each variant.

**Figure S4.** Effect of MIF-2 mutations on apparent molecular tumbling rates via *T*_1_/*T*_2_ parameters. Shown in black in each panel is the per-residue *T*_1_/*T*_2_ for wt-MIF-2, compared to a different MIF-2 variant (red). Local differences in *T*_1_ and *T*_2_ values for wt-MIF-2 and MIF-2 variants are apparent, but overall, rotational correlation time and structural compactness is preserved in MIF-2 variants. The average *T*_1_ and *T*_2_ values across all residues of wt-MIF-2 were 1154 ± 57 ms and 51 ± 5.4 ms, respectively, while those of MIF-2 variants were 1199 ± 64 ms and 51 ± 1.2 ms, respectively.

**Figure S5.** Summary of NMR *R*_1_ relaxation parameters. The per-residue *R*_1_ values are plotted for wt-MIF-2 and variants R98A, I107A, G108A, K109A, I110A and T112A. Red dashed lines represent the 1.5σ boundaries of the 10% trimmed mean of all data (blue dashed line). Overall, local differences across many of the variants differ substantially from wt-MIF-2. Most notably, there are significant dynamic changes at the C-terminal CD74 activation region caused by an F100A mutation, suggesting a coupling between these two sites. Blue dashed lines represent the 10% trimmed mean of all relaxation rates for a given parameter.

**Figure S6.** Summary of NMR *R*_2_ relaxation parameters. The per-residue *R*_2_ values are plotted for wt-MIF-2 and variants R98A, I107A, G108A, K109A, I110A and T112A. Red dashed lines represent the 1.5σ boundaries of the 10% trimmed mean of all data (blue dashed line). Overall, local differences across many of the variants differ substantially from wt-MIF-2. Most notably, there are significant dynamic changes at the C-terminal CD74 activation region caused by an F100A mutation, suggesting a coupling between these two sites. Blue dashed lines represent the 10% trimmed mean of all relaxation rates for a given parameter.

45^o^

90^o^

**Figure S7.** Regions of *R_1_* and *R_2_* dynamic perturbation in MIF-2 variants. Commonly perturbed regions by mutation have been colored as red (residues 1-60) and blue (residues 75-117) on the x-ray crystal structure of wt-MIF-2 (PDB ID: 7MSE). Variants with wt-like enzymatic activity (K109A and I110A) perturb the area colored in blue. Variants with the least wt-like activity (R98A, I107A, G108A, K109A, T112A) perturb the entire monomer, but perturbation of the red region is exclusive to these variants. Rotations of the structure show important areas of monomer-monomer contact of these regions (black arrow).

**Figure S8.** Heat map of per-residue differences in *R_1_*, *R_2_*, and ^1^H-[^15^N] NOE relaxation parameters as calculated in **Figure S10** for MIF-2 variants mapped onto the MIF-2 monomer. The blue and red colors indicate a more negative or positive value, respectively. Spectrum bars at the bottom indicate the scale associated to color intensity per parameter.

**<*σ*NOE> = 0.83**

**Figure S9.** Summary of NMR ^1^H-[^15^N] heteronuclear NOE relaxation parameters. The per-residue NOE values are plotted for wt-MIF-2 and variants R98A, I107A, G108A, K109A, I110A and T112A. Red dashed lines represent the -1.5σ boundary of the 10% trimmed mean of all data (blue dashed line). Overall, local differences across many of the variants differ substantially from wt-MIF-2. Most notably, there are significant dynamic changes at the C-terminal CD74 activation region caused by an F100A mutation, suggesting a coupling between these two sites. Blue dashed lines represent the 10% trimmed mean of all relaxation rates for a given parameter.

**Figure S10.** Per-residue differences in *R*_1_, *R*_2_, and ^1^H-[^15^] NOE relaxation parameters for MIF-2 variants. Relaxation parameters plotted for R98A, G108A, K109A, and T112A as (*R*_1_, *R*_2_, or NOE)_MUT_ – (*R*_1_, *R*_2_, or NOE)_WT_. In all panels, positive values of Δ*R*_1_ or Δ*R*_2_ indicate a greater dynamic contribution of the mutant, while negative values indicate a greater dynamic contribution of wt-MIF-2. In contrast, negative ΔNOE values indicate a greater dynamic contribution of the mutant, while positive values indicate a greater dynamic contribution of wt-MIF-2. Cartoons at top show the secondary structure elements of MIF-2 (not to scale). Horizontal dashed red lines indicate ± 1.5σ from the 10% trimmed mean of all *R*_1_, *R*_2_, and NOE data sets, respectively. Relaxation parameters that failed to reach statistical significance were defined as 0.04 ≥ Δ*R*_1_ ≤ -0.09, 2.04 ≥ Δ*R*_2_ ≤ -1.63, and 0.03 ≥ ΔNOE ≤ -0.04.

**Figure S11.** NMR order parameters derived from Model-free analysis. **(A)** Per-residue plots of *S*^2^ (top panel) and Δ*S*^2^ (calculated as *S*^2^_mutant_ – *S*^2^_wt_, bottom panel) comparing wt-MIF-2 (black dots) and I107A MIF-2 (red dots). **(B)** The same parameters are compared for wt-MIF-2 (black dots) and I110A MIF-2 (red dots).

**Figure S12.** Change in Electrostatic Eigenvector Centrality (∆EEC) upon R98A, I107A, G108A, K109A, I110A, and T112A mutations. Data are plotted as wt-mutant.

**Figure S13.** Potential interactions of the N-terminal and C-terminal allosteric residues. Residues Arg98 and Ile107 of one monomer (magenta) come in close contact with Phe2 and Ser62 of another monomer (cyan) in the crystal structure of wt-MIF-2 (PDB ID: 7MSE).

**Figure S14.** 4-Hydroxyphenylpruvate titration into wt-MIF-2 demonstrates differential binding of MIF proteins. An NMR spectral overlay of wt-MIF-2 (red) with a 1:5 molar ratio of MIF-2:HPP (blue) demonstrates chemical shift perturbations and line broadening that are mapped onto the MIF-2 structure as blue and orange spheres, respectively. Combined ^1^H^15^N chemical shift perturbations (black bars) and broadening (gray bars) caused by binding with 4-HPP. Blue and red dashed lines represent the 10% trimmed mean and 1.5σ above the mean of all shifts, respectively. Green triangles indicate 4-HPP-binding residues in MIF. 4-HPP was titrated with the molar ratio (wt-MIF-2: 4-HPP) of 1:0.5, 1:1, 1:2, and 1:5.

**Figure S15.** Representative size-exclusion chromatograms of wt-MIF-2 and MIF-2 variants (from a 26/60 Superdex 75 column). The elution peak containing pure MIF-2 is highlighted by a light orange stripe (~165 mL) in each chromatogram. The discontinuity in the chromatograms highlighted by a gray dashed line represents the beginning of fraction collection as a slight diversion of flow from the waste tubing. Also shown is SDS-PAGE gel electrophoresis of a wt-MIF-2 sample with the following lanes (L 🡪 R): Marker, A – MIF-2 prior to size exclusion chromatography, B,C – larger molecular weight species ~150 mL, D-F – pure MIF-2 eluting ~ 160 mL.
